# High-Mobility Group Box-1-Induced Angiogenesis After Indirect Bypass Surgery in a Chronic Cerebral Hypoperfusion Model

**DOI:** 10.1007/s12017-019-08541-x

**Published:** 2019-05-23

**Authors:** Shingo Nishihiro, Tomohito Hishikawa, Masafumi Hiramatsu, Naoya Kidani, Yu Takahashi, Satoshi Murai, Kenji Sugiu, Yusuke Higaki, Takao Yasuhara, Cesario V. Borlongan, Isao Date

**Affiliations:** 1 Department of Neurological Surgery, Okayama University Graduate School of Medicine, Dentistry and Pharmaceutical Science, 2-5-1, Shikata-cho, kita-ku, Okayama, 700-8558 Japan; 2 Department of Pharmaceutical Analytical Chemistry, Okayama University Graduate School of Medicine, Dentistry, and Pharmaceutical Science, 1-1-1, Tsushima naka, kita-ku, Okayama, 700-8530 Japan; 3 Department of Neurosurgery and Brain Repair, University of South Florida Morsani College of Medicine, 12901 Bruce B Downs Blvd, Tampa, FL 33612 USA

**Keywords:** Cerebral hypoperfusion, Encephalo-myo-synangiosis, High-mobility group box-1, Moyamoya disease, Vascular endothelial growth factor

## Abstract

High-mobility group box-1 (HMGB1) is a nuclear protein that promotes inflammation during the acute phase post-stroke, and enhances angiogenesis during the delayed phase. Here, we evaluated whether indirect revascularization surgery with HMGB1 accelerates brain angiogenesis in a chronic cerebral hypoperfusion model. Seven days after hypoperfusion induction, encephalo-myo-synangiosis (EMS) was performed with or without HMGB1 treatment into the temporal muscle. We detected significant increments in cortical vasculature (*p* < 0.01), vascular endothelial growth factor (VEGF) expression in the temporal muscle (*p* < 0.05), and ratio of radiation intensity on the operated side compared with the non-operated side after EMS in the HMGB1-treated group than in the control group (*p* < 0.01). Altogether, HMGB1 with EMS in a chronic hypoperfusion model promoted brain angiogenesis in a VEGF-dependent manner, resulting in cerebral blood flow improvement. This treatment may be an effective therapy for patients with moyamoya disease.

## Introduction

Moyamoya disease (MMD) is a chronic cerebral hypoperfusion state that is characterized by progressive stenosis or occlusion of the intracranial internal carotid artery (Kuroda and Houkin 2008). For patients with MMD, various treatments such as direct or indirect revascularization surgery are performed in clinical practice since 40 years ago (Karasawa et al. 1977, 1978; Park et al. 2018; Deng et al. 2018). Encephalo-myo-synangiosis (EMS) is often used as indirect revascularization surgery for pediatric patients with MMD. EMS is a simple technique, but the procedure sometimes supplies insufficient collateral flow, but not enough to prevent ischemic stroke (Kuroda and Houkin 2008; Mizoi et al. 1996; Kim et al. 2010). We previously examined the effect of indirect revascularization surgery with vascular endothelial growth factor (VEGF) gene administration into the temporal muscle in a chronic cerebral hypoperfusion state in a rat model (Kusaka et al. 2005; Katsumata et al. 2010). Moreover, we investigated the effect of combined gene therapy with VEGF plus apelin during indirect revascularization surgery (Hiramatsu et al. 2017). EMS for the hypoperfusion state after bilateral common carotid artery (CCA) ligation simulated indirect bypass surgery for patients with MMD. These results indicate that EMS with combined gene therapy significantly enhanced angiogenesis in the brain cortex compared to EMS alone.

High-mobility group box-1 (HMGB1) is a non-histone nuclear DNA-binding protein that is present in most eukaryotic cells, including neural cells, to stabilize nucleosome formation, and it is related to gene expression such as gene transcription, replication, and DNA repair (van Beijnum et al. 2008; Hayakawa et al. 2010; Sama et al. 2004). HMGB1 increases inflammation and necrosis in the acute phase after stroke, but promotes neurogenesis and angiogenesis in the delayed phase after stroke (Hayakawa et al. 2010; Yang et al. 2014). There were several reports that HMGB1 is related to enhanced angiogenesis, in a hindlimb ischemic model (Biscetti et al. 2010), in a cardiac ischemic model (Kitahara et al., 2008), and in a rat model of intracerebral hemorrhage (Lei et al. 2013, 2015).

In this study, we investigated whether EMS combined with HMGB1 administration promotes brain angiogenesis in a chronic cerebral hypoperfusion model in rats. Additionally, we examined whether brain angiogenesis contributed to improving cerebral perfusion.

## Materials and Methods

### Ethics Statement

All experimental procedures performed in this study were conducted in accordance with the Institutional Animal Care and Use Committee of Okayama University Graduate School of Medicine, Dentistry and Pharmaceutical Sciences.

### Operative Procedure

Adult male Wistar rats (*n* = 63, 9 week old; SHIMIZU Laboratory Supplies Co., Ltd., Kyoto, Japan) were anesthetized with 3% sevoflurane in a mixture of 40% oxygen and 60% nitrous oxide. Under general anesthesia, the rats were placed in the supine position and bilateral CCAs were exposed after ventrocervical incision. Then, after carefully separating the CCAs from the sympathetic and vagal nerves, CCAs were doubly ligated with 3–0 silk sutures. The body temperature was maintained about 37 °C throughout the procedure using a heating pad.

Seven days after CCA occlusion, all the rats were placed in the prone positioned in a stereotactic apparatus following general anesthesia administered via an intraperitoneal injection (0.5 ml/100 g, medetomidine, 7.5%; midazolam, 8%; butorphanol, 10%; saline, 74.5%). The right temporal muscle was detached from the temporal bone after a midline incision. After a right-sided craniectomy was performed in the temporoparietal region using a dental drill, the dura mater was carefully removed from the brain surface without damaging the brain tissue. The exposed brain surface was covered with the temporal muscle in all the rats. A total of 1 µg of HMGB1 (HMGBiotech, Milano, Italy) in 0.1 mL of phosphate-buffered saline (PBS) was slowly injected into the detached temporal muscle at three different sites using a microsyringe in 5–10 min in order to not overflow from the temporal muscle. In the control group, all the procedures were performed with administration of an equivalent amount volume of 0.9% saline, but not HMGB1 solution. After injection, the skin was closed using 3–0 silk sutures. Before and after the procedures, all rats were kept in a temperature- and humidity-controlled room, and had free access to food and water.

### Immunohistochemical Analysis

The rats were deeply anesthetized and perfused with 200 mL of PBS, followed by 100 mL of 4% paraformaldehyde (PFA) 4 days and 14 days after EMS surgery with or without HMGB1 administration. The whole brain was harvested and fixed for 24 h in 4% PFA, and embedded in paraffin. Coronal sections (4.5 μm) were cut from each specimen using a cryostat, and the sections were mounted on slides. Two coronal sections that were in the anterior and posterior parts below the EMS surgery were prepared from each animal (control group *n* = 5, HMGB1-treated group *n* = 5 at 4 days; control group *n* = 5, HMGB1-treated group *n* = 6 at 14 days).

For immunofluorescent staining of endothelial cells, the slides were soaked twice in limonene for 10 min each, twice in 100% alcohol for 5 min each, and twice in 90% alcohol for 5 min each for deparaffinization. The slides were soaked in citrate buffer (pH 6.0) that was autoclaved at 121 °C for 15 min for antigen retrieval. The slides were then incubated with 10% normal goat serum in PBS for 1 h at room temperature. The slides were incubated with primary antibody (rabbit anti-CD31 antibody, 1:50; Abcam, ab28364) overnight at 4 °C. The slides were washed and incubated with secondary antibody (Alexa Fluor 488 anti-rabbit IgG antibody, 1:250; Abcam, ab150077) for 1 h at room temperature.

On each slide, three different fields in the cortex both of two different sections at 3 and 7 mm posterior to the bregma, under the covered temporal muscle on the operated side and on the non-operated side were randomly photographed, and the images were analyzed by a blinded examiner using ImageJ software (National Institutes of Health, Bethesda, USA). The number of vessels per field was calculated automatically, and the number of vessels in each cortex was expressed as the average number of all fields on each side.

### Enzyme-Linked Immunosorbent Assay Analysis of VEGF

Four days and 14 days after EMS surgery, brains and temporal muscles were quickly harvested after decapitation with overdosed pentobarbital (150 mg/kg, intraperitoneal). Thirty milligrams each of brain cortex and muscles tissue was obtained. Brain tissue was homogenized using N-PER Tissue Protein Extraction Reagent (Thermo Fisher Scientific Inc., Massachusetts, USA), and muscles were in N-PER Neuronal Protein Extraction Reagent (Thermo Fisher Scientific Inc., Massachusetts, USA). They were centrifuged at 10,000×*g* for 10 min at 4 °C, and the supernatant was obtained. One hundred microliters of the samples were applied to dish and VEGF protein level in each tissue (control group *n* = 7, HMGB1-treated group *n* = 7 at 4 days; control group *n* = 9, HMGB1-treated group *n* = 8 at 14 days) was measured using a rat VEGF ELISA assay kit (#27101 Rat VEFG Assay Kit, Immuno-Biological Laboratories Co., Ltd., Gunma, Japan) according to the manufacturer’s instructions.

### Cerebral Blood Flow Assessment

Fourteen days after EMS surgery and CCA occlusion, the rats (control group *n* = 7, HMGB1-treated group *n* = 8) were scanned using a small animal SPECT with *N*-isopropyl-^123^I-p-iodoamphetamine (^123^I-IMP; Iofetamine Injection Daiichi, FUJIFILM RI Pharma Co., Ltd., Tokyo, Japan) tracer to evaluate CBF. The rats were placed in the prone position after general anesthesia with 3% sevoflurane in a mixture of room air (flow, 1.0–2.0 L/min). The scan was performed under general anesthesia 15 min after ^123^I-IMP tracer injection (30 MBq) into the lateral tail vein.

The images were obtained using the SPECT/CT scanner (FX3000, TriFoil Imaging Inc., Northridge, CA, USA) with cadmium–zinc–telluride semiconductors and multi- pinhole collimators (focal length, 65 mm; aperture, 1.0 mm) after CT acquisition. The images were reconstructed using FLEX-RECON software with a three-dimensional ordered subset expectation maximization (3D-OSEM; iteration, 5; subset, 8) algorithm, followed by data collection (360° acquisition, 30 s/frame, 64 frames/total) with 45-mm semidiameter detectors.

**Fig. 1:**
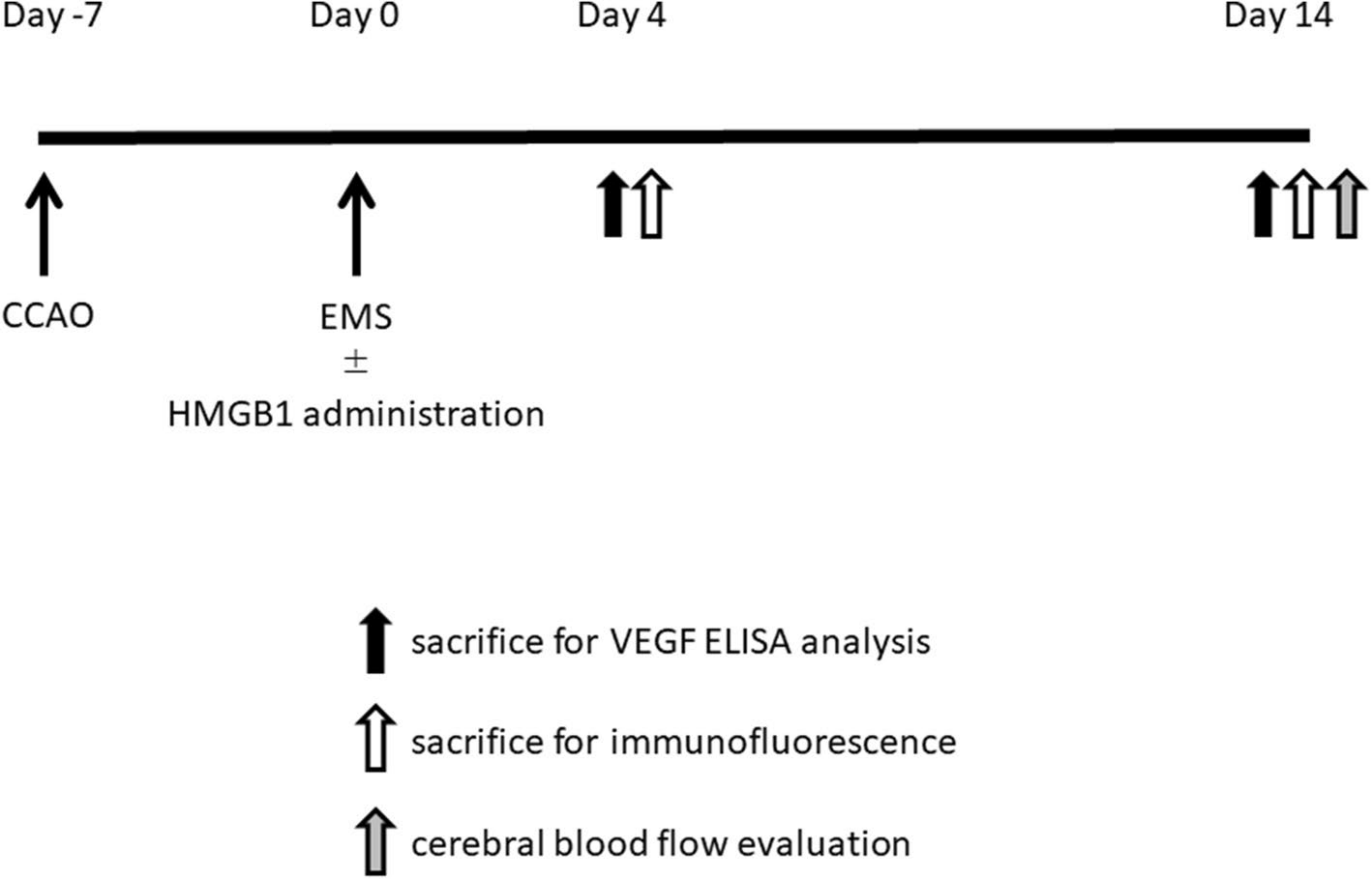
Experimental schedule. The rats underwent bilateral common carotid artery (CCAs) occlusion. Seven days after CCA occlusion, encephalo-myo-synangiosis (EMS) with or without HMGB1 administration into the temporal muscle was performed. Immunohistochemical analysis and vascular endothelial growth factor (VEGF) analysis were performed 4 and 14 days after EMS. Cerebral blood flow was assessed 14 days after EMS

CBF imaging was automatically obtained using analysis software (AMIDE, a Medical Imaging Data Examiner version 1.0.5), and analyzed semiquantitatively. CBF analysis was performed using two different anterior and posterior coronal slices including the operated area. CBF in the cortex on both sides was measured using the region of interest (ROI) settings. Each ROI was a circle with a diameter of 2 mm, which was placed on the cortex. In each slice, the ROI was placed in three fields on the cortex under the covered temporal muscle on the operated side, and the same placement was made on the non-operated side. The radiation intensity of each ROI was calculated automatically, and that of each cortex was expressed as the average intensity of all ROIs on each side. The perfusion ratio was expressed as a percentage obtained using the following formula: average radiation intensity on the operated side/average radiation intensity on the non-operated side. The schedule for the experiments is presented in Fig. 1.

### Statistical Analysis

All data in the study are presented as the mean ± SD. Data were analyzed using a one-way analysis of variance (ANOVA) with a post hoc Turkey analysis or student’s *t* test using SPSS statistical software, version 20 (SPSS, Inc., Chicago, IL, USA). A *p* value less than 0.05 was considered to be statistically significant.

## Results

### Number of Blood Vessels in the Brain Cortex

**Fig. 2:**
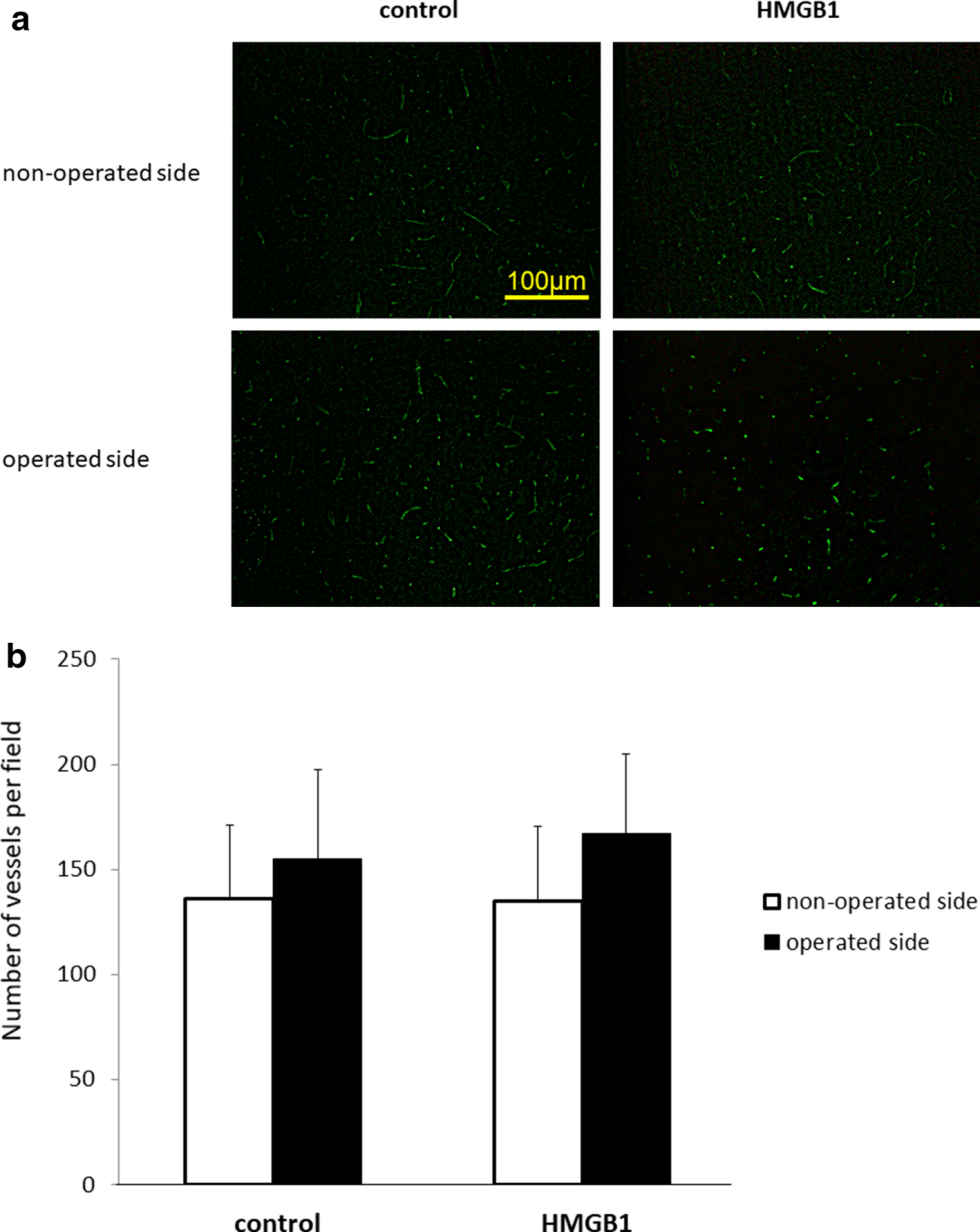
The number of blood vessels in the brain cortex 4 days after EMS. **a** Representative photomicrographs of the control and HMGB1-treated groups stained with antibody against CD31. **b** The number of vessels in the brain cortex 4 days after EMS was not statistically different on both sides between the two groups

**Fig. 3:**
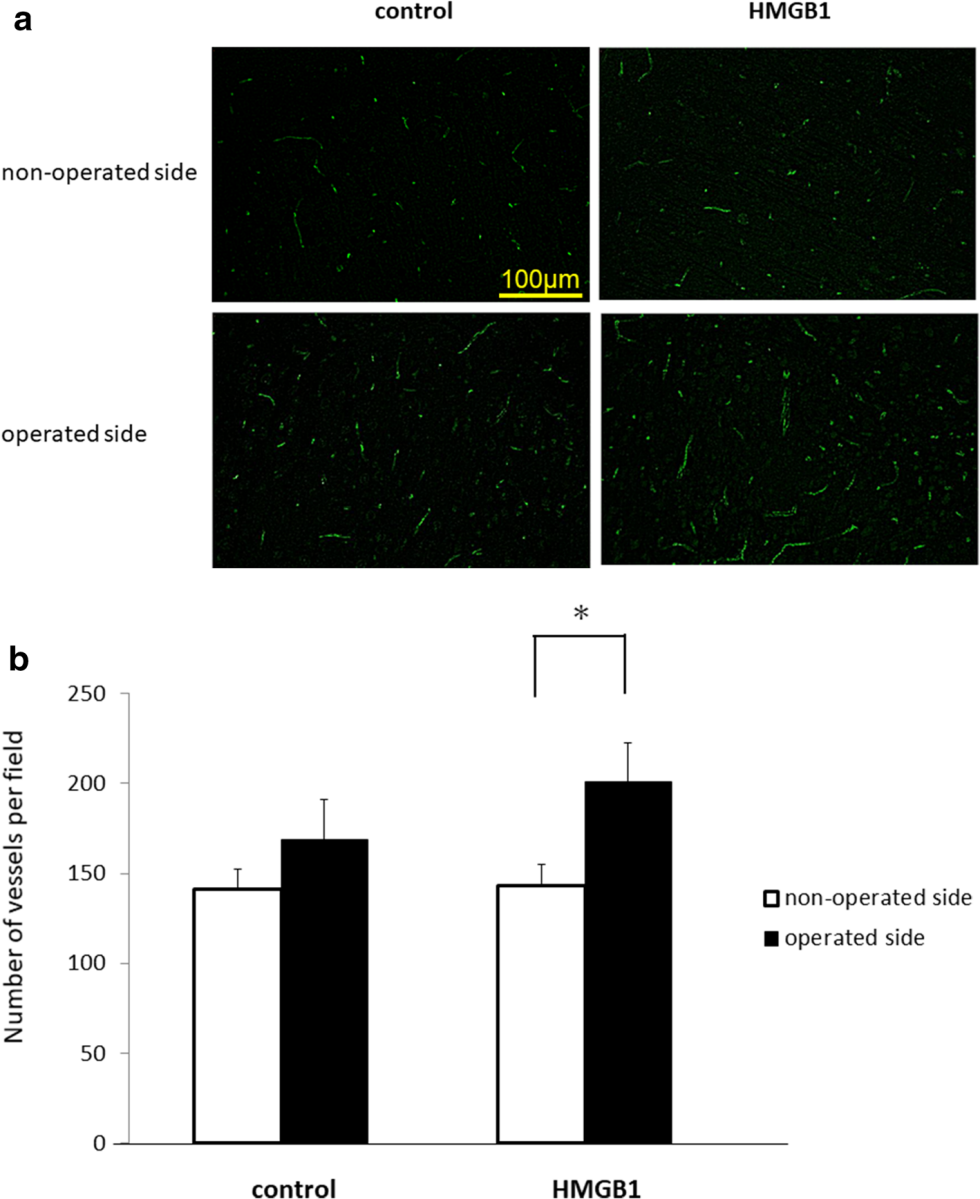
Number of blood vessels in the brain cortex 14 days after EMS. **a** Representative photomicrographs of the control- and HMGB1-treated groups stained with antibody against CD31. **b** The number of vessels in the brain cortex on the operated side in the HMGB1-treated group was higher than that on the operated side in the control group, and it was significantly higher compared to the non-operated side in the HMGB1-treated group (*p < 0.01)

The number of vessels in the brain cortex 4 days after EMS showed no difference on both sides between the two groups (control: 137 ± 23/155 ± 35, HMGB1: 132 ± 18/166 ± 15, (non-operated side/operated side), Fig. 2a and b, *p* = 0.874, operated side of control versus that of HMGB1-treated group). However, 14 days after EMS, the number of vessels in the brain cortex on the operated side in the HMGB1-treated group (201 ± 23) tended to be higher than that on the operated side in the control group (169 ± 25, *p* = 0.069, control vs. HMGB1-treated group) and was significantly higher than the non-operated side in the HMGB1-treated group (143 ± 13, Fig. 3a and b, *p* < 0.01).

### Expression of VEGF Protein Levels in Brain Cortex and Temporal Muscle

**Fig. 4:**
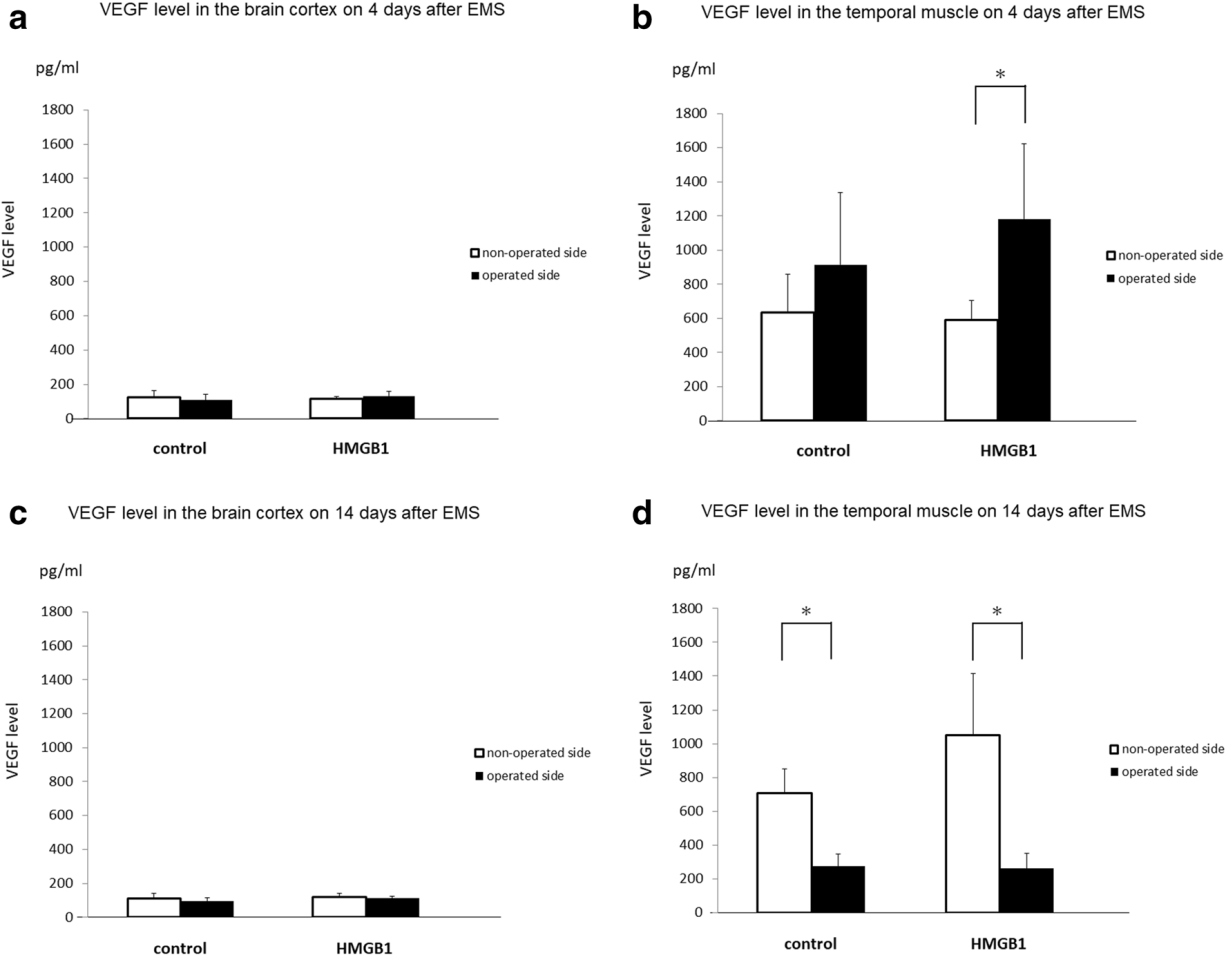
Expression of VEGF protein levels in the brain cortex and temporal muscle. **a** VEGF protein expression in the brain cortex 4 days after EMS was not statistically different on both sides between the two groups. **b** In the temporal muscle, VEGF expression on the operated side in the HMGB1-treated group was higher than that of the operated side in the control group and was significantly higher compared to the non-operated side in the same group 4 days after EMS (*p < 0.05). **c** VEGF expression in the brain cortex 14 days after EMS was not statistically different on both sides between the two groups. **d** In the temporal muscle, VEGF expression on the operated side in both groups was significantly decreased compared to the non-operated side 14 days after EMS (*p < 0.01, **p < 0.01)

The VEGF expression level in the brain cortex 4 days after EMS revealed no difference on both sides between the two groups (control: 126 ± 39/108 ± 34, HMGB1: 114 ± 14/130 ± 32 pg/ml, (non-operated side/operated side), Fig. 4a). In the temporal muscle, however, VEGF expression on the operated side in HMGB1-treated group (1182 ± 474 pg/ml) was not significantly different from that on the operated side in the control group (915 ± 454 pg/ml, *p* = 0.507, control vs. HMGB1-treated group) and was significantly higher compared with the non-operated side in the HMGB1-treated group (589 ± 125), Fig. 4b, *p* < 0.05). The VEGF expression level in the brain cortex 14 days after EMS revealed no difference in both sides between the two groups (control: 109 ± 32/93 ± 23, HMGB1: 120 ± 21/111 ± 16 pg/ml, (non-operated side/operated side), Fig. 4c). In the temporal muscle, however, VEGF expression on the operated side in both groups (control: 275 ± 74, HMGB1: 262 ± 95 pg/ml) was significantly decreased compared to those on the non-operated side (control: 707 ± 153, HMGB1: 1052 ± 388 pg/ml) 14 days after EMS (Fig. 4d, *p* < 0.01).

### SPECT Analysis

**Fig. 5:**
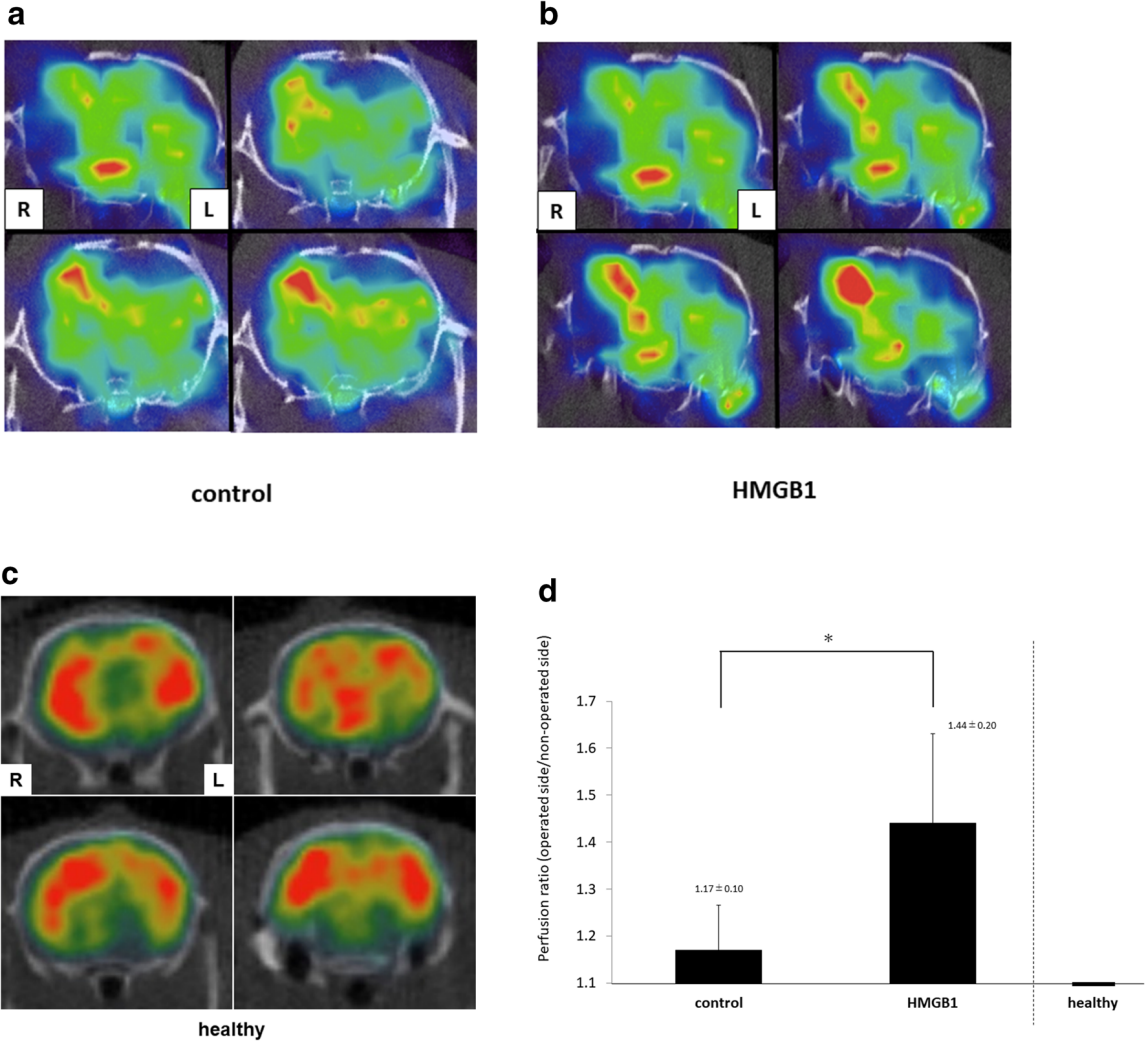
Cerebral blood flow 14 days after EMS. **a**–**c** Representative SPECT image obtained 14 days after EMS showing improved cerebral perfusion in both groups (A: control, B: HMGB-1, C: healthy). **d** The ratio of radiation intensity on the operated side compared to the non-operated side was significantly higher in the HMGB1-treated group than that in the control group (*p < 0.01). The healthy rats show no apparent laterality of cerebral blood flow

SPECT analysis obtained 14 days after EMS showed that cerebral blood flow in the brain cortex below EMS increased compared with the contralateral brain cortex in both groups (Fig. 5a and b, healthy rats: Fig. 5c). The ratio of radiation intensity on the operated side compared with the non-operated side was significantly higher in the HMGB1-treated group (1.44 ± 0.2) than that in the control group (1.17 ± 0.1, Fig. 5d, *p* < 0.01).

## Discussion

### Bypass Surgery for Moyamoya Disease

Direct bypass surgery such as STA-MCA bypass is frequently performed for adult patients with MMD (Karasawa et al. 1978; Mizoi et al. 1996), but the procedure is sometimes difficult to perform especially in pediatric patients. Thus, indirect bypass surgery such as EMS is often chosen for surgical treatment of pediatric patients. Although indirect bypass surgery is an easy and effective treatment for patients with MMD, it may sometimes supply insufficient collateral flow, resulting in a poor clinical outcome (Mizoi et al. 1996; Kim et al. 2010; Hayakawa et al. 2010; Pandey and Steinberg 2011). The postoperative clinical outcome depends on brain angiogenesis because of transmuscular anastomosis crossing from the covered temporal muscle and the improvement in CBF (Cho et al. 2014; Ishii et al. 2017).

Exogenous angiogenic factors administered during indirect bypass surgery may increase collateral circulation, which leads to an improvement in CBF. Previous experimental reports showed that administration of the plasmid human VEGF with EMS significantly increased the number of vessels compared with EMS surgery alone (Kusaka et al. 2005; Katsumata et al. 2010). Moreover, in the combination of gene therapy with VEGF plus apelin, the number of vessels was significantly increased compared with EMS alone, and mature vessels were significantly developed compared with EMS alone, or compared with administration of plasmid human VEGF with EMS (Hiramatsu et al. 2017). These data suggest that administration of other angiogenic factors with EMS surgery may also enhance angiogenesis, leading to an improvement in cerebral circulation.

### Angiogenic Effect of HMGB1 in a Chronic Cerebral Hypoperfusion State

HMGB1 is a non-histone nuclear DNA-binding protein that is widely observed in most eukaryotic cells including neural cells, and it is involved in gene expression such as gene transcription, replication, and DNA repair(van Beijnum et al. 2008; Hayakawa et al. 2010; Sama et al. 2004). In the early phase after brain injury and stroke, HMGB1 is passively secreted from damaged or necrotic cells and it is actively secreted from immune cells, inducing inflammation and necrosis (Hayakawa et al. 2010; Scaffidi et al. 2002). However, in the late phase after neural ischemia, HMGB1 may enhance angiogenesis resulting in tissue remodeling by endothelial activation and sprouting, (Yang et al. 2014) and it may promote neurovascular repair by activating endothelial progenitor cells after cerebral ischemia (Hayakawa et al. 2012).

Injection of HMGB1 into the ischemic hindlimb of diabetic mice induced angiogenesis and promoted perfusion recovery via a VEGF-dependent mechanism (Biscetti et al. 2010). Enhanced angiogenesis and improved cardiac function were shown in HMGB1 transgenic mice using a left anterior-descending coronary artery ligation mouse model (Kitahara et al. 2008). In the present chronic cerebral hypoperfusion model in rats, the number of vessels in the cortex on the operated side in the HMGB1-treated group was higher compared with the same side in the control group, and was significantly higher compared with the non-operated side in the same group 14 days after EMS. Additionally, we revealed that the VEGF expression level in muscle on the operated side in the HMGB1-treated group was significantly higher compared with the non-operated side in the same group 4 days after EMS, while the expression level of VEGF in the cortex showed no difference on both sides between the two groups. HMGB1 directly increased vessel formation through promoting endothelial cell proliferation and migration, and indirectly increased vessel formation by promoting angiogenic factors such as VEGF (Hayakawa et al. 2012). Thus, HMGB1 could promote the production of angiogenic cytokines such as VEGF from endothelial cells and activated macrophages.

HMGB1-induced angiogenesis as a function of VEGF has been examined in diabetic mice (Biscetti et al. 2010). HMGB1 administration promotes ischemia-induced angiogenesis in a VEGF-dependent manner (0–800 ng) in these diabetic mice (Biscetti et al. 2010). In our preliminary study, 1 µg of HMGB1 showed strong angiogenic potentials, compared to less amount of HMGB1. Thus, we determined the dose of HMGB1 used in this study. Additionally, we observed that the VEGF protein was significantly increased in the muscle on the operated side, and significantly promoted angiogenesis in the cortex of the operated side in the HMGB1-treated group. This indicates that angiogenesis in the cortex was enhanced followed by transmuscular anastomosis that developed into the brain cortex below EMS through the VEGF-dependent mechanism (Kusaka et al. 2005). Additionally, angiogenesis in the cortex on the operated side in the HMGB1-treated group occurred 14 days after EMS surgery, while VEGF expression in the muscle on the operated side in the HMGB1-treated group was elevated 4 days after EMS surgery in this investigation. The duration of angiogenesis in this study was 14 days after EMS surgery, which was consistent with results of previous studies (Kusaka et al. 2005; Hiramatsu et al. 2017). The VEGF level was elevated a few days after HMGB1 administration into the ischemic hindlimb of diabetic mice, and enhanced post-ischemic angiogenesis due to HMGB1 administration was confirmed at least 7 days after ischemia, when the tissue recovery and the inflammatory response were comparable (Biscetti et al. 2010). VEGF expression in the muscle on the operated side decreased because angiogenesis was enhanced followed by developed transmuscular anastomosis below EMS, which could contribute to improvement of the ischemic condition in the brain cortex.

In previous reports, the direct injection of VEGF protein into the lateral ventricle (Harrigan et al. 2002) or gene therapy using a vehicle such as a virus or plasmid (Kusaka et al. 2005; Harrigan et al. 2002) is performed to enhance angiogenesis. However, in newly formed immature vessels produced by VEGF alone, side effects such as edema (Harrigan et al. 2002; Baumgartner et al. 2000) or angioma formation (Schwarz et al. 2000) may be a concern. Gene therapy using a virus or plasmid may not be safe for direct injection into the patients with MMD. In the gene therapy, the targeted genes may be inserted into the wrong point in the host DNA, which leads to DNA mutations, and the cells proliferate abnormally, resulting in tumor formation. HMGB1 administration into the temporal muscle, however, is a superior treatment compared with the gene therapy in terms of safety, especially to reduce DNA mutations. This method is simple and effective because only a single HMGB1 administration could promote production of angiogenic factors such as VEGF, which leads to angiogenesis, and also directly promotes angiogenesis through stimulating endothelial cell proliferation and migration (Yang et al. 2014). In addition to angiogenesis, HMGB1 plays an important role in regeneration of the nervous system (Rong et al. 2004).

### SPECT Image

In clinical practice, improvement in CBF after direct or indirect bypass surgery leads to the prevention of ischemic stroke for patients with MMD (Kuroda and Houkin^2008^). This suggests that hemodynamic improvement of cerebral circulation after revascularization surgery is crucial for the postoperative outcome (So et al. 2005; Kim et al. 2004). Hecht et al. (2015) evaluated the recovery of cerebrovascular reserve capacity after an indirect revascularization procedure with VEGF-expressing myoblasts using laser speckle imaging (Hecht et al. 2015).

In our experiment, we also performed molecular imaging to evaluate the therapeutic effect on cerebral perfusion after revascularization surgery. CBF in the cortex after indirect revascularization surgery was investigated in vivo using SPECT to confirm the effect of angiogenesis. We found that the ratio of cerebral perfusion (operated side/non-operated side) in the HMGB1-treated group was elevated more than that in the control group, and this evaluation was significant. This result suggests that administration of HMGB1 with EMS could significantly improve cerebral perfusion through angiogenesis after indirect revascularization surgery compared to EMS alone. This study is the first report to reveal that the number of vessels in the cortex was increased by angiogenesis, and also to use SPECT imaging to show the recovery of cerebral perfusion after indirect revascularization surgery for a chronic cerebral hypoperfusion state. Additionally, improvement of CBF after indirect revascularization surgery was observed sequentially using in vivo SPECT imaging.

### Study Limitations

This study was designed to evaluate the effect of indirect bypass surgery with HMGB1 administration, but our experiment has several limitations.

First, the immunohistochemical analysis period and the CBF study were short. In clinical practice, angiogenesis and collateral formation originate much later, after indirect bypass surgery in patients with MMD. Therefore, vessel formation, protein expression, and CBF need to be observed over a longer period.

Second, we did not evaluate vessel function, such as collapse of the blood–brain barrier, vessel leakage, and brain edema which are affected by the permeability-enhancing effect of VEGF.

Third, we did not perform behavioral and cognitive function assessment before and after treatment. The impaired status, such as cognitive dysfunction, before treatment may be improved because of the development of cerebral circulation after the treatment, which requires further study. It is important to evaluate whether behavioral and cognitive function are improved in association with the improvement of CBF after the treatment.

## Conclusion

HMGB1 administration with EMS surgery in a chronic hypoperfusion rat model promotes angiogenesis through a VEGF-dependent mechanism, and this enhanced angiogenesis likely contributes to improved cerebral circulation. These results support HMGB1 as a novel therapeutic approach for patients with MMD.

## References

[CR1] Baumgartner I, Rauh G, Pieczek A, Wuensch D, Magner M, Kearney M (2000). Lower-extremity edema associated with gene transfer of naked DNA encoding vascular endothelial growth factor. Annals of Internal Medicine.

[CR2] Biscetti F, Straface G, De Cristofaro R, Lancellotti S, Rizzo P, Arena V (2010). High-mobility group box-1 protein promotes angiogenesis after peripheral ischemia in diabetic mice through a VEGF-dependent mechanism. Diabetes.

[CR3] Cho WS, Kim JE, Kim CH, Ban SP, Kang HS, Son YJ (2014). Long-term outcomes after combined revascularization surgery in adult moyamoya disease. Stroke.

[CR4] Deng X, Gao F, Zhang D, Zhang Y, Wang R, Wang S (2018). Effects of different surgical modalities on the clinical outcome of patients with moyamoya disease: A prospective cohort study. Journal of Neurosurgery.

[CR5] Harrigan MR, Ennis SR, Masada T, Keep RF (2002). Intraventricular infusion of vascular endothelial growth factor promotes cerebral angiogenesis with minimal brain edema. Neurosurgery.

[CR6] Hayakawa K, Qiu J, Lo EH (2010). Biphasic actions of HMGB1 signaling in inflammation and recovery after stroke. Annals of the New York Academy of Sciences.

[CR7] Hayakawa K, Pham LD, Katusic ZS, Arai K, Lo EH (2012). Astrocytic high-mobility group box 1 promotes endothelial progenitor cell-mediated neurovascular remodeling during stroke recovery. Proceedings of the National Academy of Sciences of the United States of America.

[CR8] Hecht N, Marushima A, Nieminen M, Kremenetskaia I, von Degenfeld G, Woitzik J (2015). Myoblast-mediated gene therapy improves functional collateralization in chronic cerebral hypoperfusion. Stroke.

[CR9] Hiramatsu M, Hishikawa T, Tokunaga K, Kidoya H, Nishihiro S, Haruma J (2017). Combined gene therapy with vascular endothelial growth factor plus apelin in a chronic cerebral hypoperfusion model in rats. Journal of Neurosurgery.

[CR10] Ishii Y, Tanaka Y, Momose T, Yamashina M, Sato A, Wakabayashi S (2017). Chronologic evaluation of cerebral hemodynamics by dynamic susceptibility contrast magnetic resonance imaging after indirect bypass surgery for moyamoya disease. World Neurosurgery.

[CR11] Karasawa J, Kikuchi H, Furuse S, Sakaki T, Yoshida Y (1977). A surgical treatment of “moyamoya” disease “encephalo-myo synangiosis”. Neurologia Medico-chirurgica.

[CR12] Karasawa J, Kikuchi H, Furuse S, Kawamura J, Sakaki T (1978). Treatment of moyamoya disease with STA-MCA anastomosis. Journal of Neurosurgery.

[CR13] Katsumata A, Sugiu K, Tokunaga K, Kusaka N, Watanabe K, Nishida A (2010). Optimal dose of plasmid vascular endothelial growth factor for enhancement of angiogenesis in the rat brain ischemia model. Neurol Med Chir (Tokyo).

[CR14] Kim SK, Seol HJ, Cho BK, Hwang YS, Lee DS, Wang KC (2004). Moyamoya disease among young patients: Its aggressive clinical course and the role of active surgical treatment. Neurosurgery.

[CR15] Kim SK, Cho BK, Phi JH, Lee JY, Chae JH, Kim KJ (2010). Pediatric moyamoya disease: An analysis of 410 consecutive cases. Annals of Neurology.

[CR16] Kitahara T, Takeishi Y, Harada M, Niizeki T, Suzuki S, Sasaki T (2008). High-mobility group box 1 restores cardiac function after myocardial infarction in transgenic mice. Cardiovascular Research.

[CR17] Kuroda S, Houkin K (2008). Moyamoya disease: current concepts and future perspectives. Lancet Neurol.

[CR18] Kusaka N, Sugiu K, Tokunaga K, Katsumata A, Nishida A, Namba K (2005). Enhanced brain angiogenesis in chronic cerebral hypoperfusion after administration of plasmid human vascular endothelial growth factor in combination with indirect vasoreconstructive surgery. Journal of Neurosurgery.

[CR19] Lei C, Lin S, Zhang C, Tao W, Dong W, Hao Z (2013). Effects of high-mobility group box1 on cerebral angiogenesis and neurogenesis after intracerebral hemorrhage. Neuroscience.

[CR20] Lei C, Zhang S, Cao T, Tao W, Liu M, Wu B (2015). HMGB1 may act via RAGE to promote angiogenesis in the later phase after intracerebral hemorrhage. Neuroscience.

[CR21] Mizoi K, Kayama T, Yoshimoto T, Nagamine Y (1996). Indirect revascularization for moyamoya disease: Is there a beneficial effect for adult patients?. Surgical Neurology.

[CR22] Pandey P, Steinberg GK (2011). Outcome of repeat revascularization surgery for moyamoya disease after an unsuccessful indirect revascularization. Clinical article. Journal of Neurosurgery.

[CR23] Park SE, Kim JS, Park EK, Shim KW, Kim DS (2018). Direct versus indirect revascularization in the treatment of moyamoya disease. Journal of Neurosurgery.

[CR24] Rong LL, Trojaborg W, Qu W, Kostov K, Yan SD, Gooch C (2004). Antagonism of RAGE suppresses peripheral nerve regeneration. FASEB J.

[CR25] Sama AE, D’Amore J, Ward MF, Chen G, Wang H (2004). Bench to bedside: HMGB1-a novel proinflammatory cytokine and potential therapeutic target for septic patients in the emergency department. Academic Emergency Medicine.

[CR26] Scaffidi P, Misteli T, Bianchi ME (2002). Release of chromatin protein HMGB1 by necrotic cells triggers inflammation. Nature.

[CR27] Schwarz ER, Speakman MT, Patterson M, Hale SS, Isner JM, Kedes LH, Kloner RA (2000). Evaluation of the effects of intramyocardial injection of DNA expressing vascular endothelial growth factor (VEGF) in a myocardial infarction model in the rat–angiogenesis and angioma formation. Journal of the American College of Cardiology.

[CR28] So Y, Lee HY, Kim SK, Lee JS, Wang KC, Cho BK (2005). Prediction of the clinical outcome of pediatric moyamoya disease with postoperative basal/acetazolamide stress brain perfusion SPECT after revascularization surgery. Stroke.

[CR29] van Beijnum JR, Buurman WA, Griffioen AW (2008). Convergence and amplification of toll-like receptor (TLR) and receptor for advanced glycation end products (RAGE) signaling pathways via high mobility group B1 (HMGB1). Angiogenesis.

[CR30] Wang YQ, Guo X, Qiu MH, Feng XY, Sun FY (2007). VEGF overexpression enhances striatal neurogenesis in brain of adult rat after a transient middle cerebral artery occlusion. Journal of Neuroscience Research.

[CR31] Yang S, Xu L, Yang T, Wang F (2014). High-mobility group box-1 and its role in angiogenesis. Journal of Leukocyte Biology.

